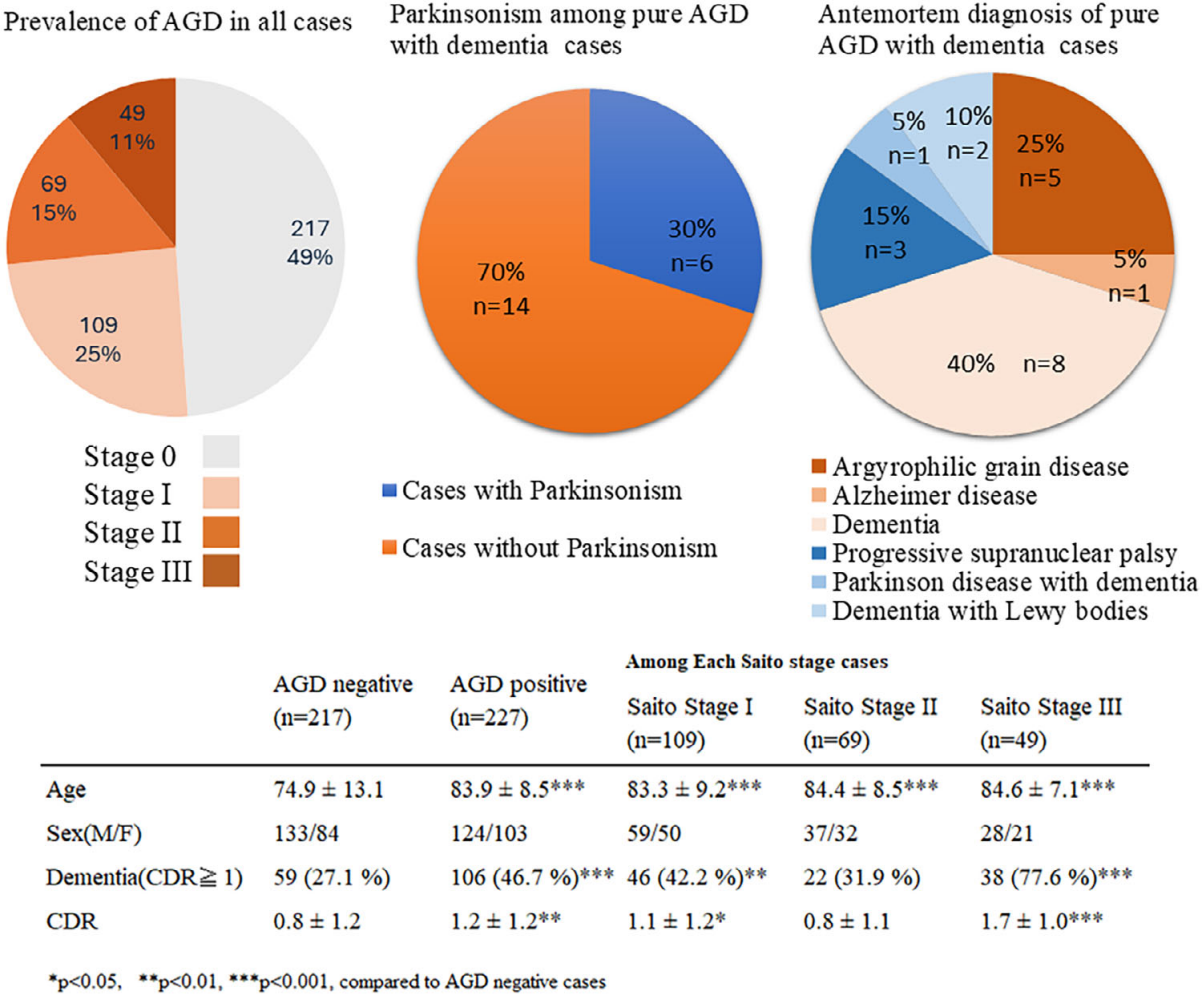# Prevalence of argyrophilic grain disease and its clinical impact on cognitive decline and parkinsonism in a consecutive autopsy cohort in Japan

**DOI:** 10.1002/alz70857_099998

**Published:** 2025-12-24

**Authors:** Akira Arakawa, Tomoyasu Matsubara, Manato Hara, Yuko Saito, Shigeo Mutayama

**Affiliations:** ^1^ Brain Bank for Aging Research, Tokyo Metropolitan Geriatric Hospital and Institute of Gerontology, Tokyo, Tokyo, Japan; ^2^ The Brain Bank for Neurodevelopmental, Neurological and Psychiatric Disorders, United Graduate School of Child Development, Graduate School of Medicine, Osaka University, Osaka, Osaka, Japan

## Abstract

**Background:**

Argyrophilic grain disease (AGD) is an age‐related disorder characterized by the presence of argyrophilic grains. AGD has sequential distribution pattern (Saito stage). The main clinical features involve amnestic dementia although conflicting results are reported on the association with dementia. Recent studies also suggest its association with parkinsonism. To clarify the association of AGD with dementia and parkinsonism, we performed clinicopathological study using the Brain Bank for Aging Research autopsy cohort in Japan.

**Method:**

We retrospectively examined the age, Saito stage, and CDR of 452 consecutive autopsy cases of BBAR from October 2012 to September 2022. We contracted pure AGD with dementia cases; cases with dementia (CDR ≧ 1) and no other comorbid pathology, namely, Braak stage ≤ 2, BBAR Lewy stage ≤ 1 were selected, and antemortem diagnosis and clinical features were examined retrospectively from the medical records.

**Result:**

About half, 227 of the cases of the 452 consecutive autopsy cases had AGD and the number of cases was 109, 69, 49 for Saito stage I, II, III respectively. The frequency of AGD and average Saito stage increased with age. The rate of demented cases and average CDR score were significantly higher in AGD positive cases (46.7% and 1.2 ± 1.2) than AGD negative cases (27.1% and 0.8 ± 1.2). The rate of demented cases and average CDR score were significantly higher in Saito stage III cases (77.6% and 1.7 ± 1.0) and Saito stage I cases (42.2% and 1.1 ± 1.2). The rate and score tended to be higher but with no significance in Saito stage II cases (31.9% and 0.8 ± 1.1). Twenty cases were diagnosed as pure AGD with dementia. Six among 20 cases presented with parkinsonism, especially postural instability in addition to amnestic dementia, and diagnosed as progressicve supranuclear palsy (*n* = 3), Parkinson disease with dementia (*n* = 1) and dementia with Lewy bodies (*n* = 2).

**Conclusion:**

AGD has a strong association with dementia especially for Saito Stage III cases and parkinsonism may be a common clinical presentation of AGD.